# Spatial proteomics reveals phenotypic and functional differences in T cell and macrophage subsets during villitis of unknown etiology

**DOI:** 10.1038/s41598-024-51545-2

**Published:** 2024-01-09

**Authors:** Petra K. Lothert, Bohdana Fedyshyn, Sylvie Girard, Rana Chakraborty, Andrew P. Norgan, Elizabeth Ann L. Enninga

**Affiliations:** 1 Mayo Clinic Graduate School of Biomedical Sciences, Mayo Clinic, Rochester, MN USA; 2 Department of Immunology, Mayo Clinic, Rochester, MN USA; 3 Department of Obstetrics and Gynecology, Mayo Clinic, Rochester, MN USA; 4 Department of Pediatric and Adolescent Medicine, Mayo Clinic, Rochester, MN USA; 5 Department of Laboratory Medicine and Pathology, Mayo Clinic, Rochester, MN USA

**Keywords:** Immunology, Chronic inflammation

## Abstract

Villitis of unknown etiology (VUE) is a prevalent inflammatory pathology of the placenta characterized by infiltration of maternal T cells and accumulation of fetal macrophages into chorionic villi. VUE is associated with a variety of adverse clinical outcomes, including fetal growth restriction and fetal demise. Evaluation of the phenotypic and functional differences between two immune cell types associated with this pathology, namely T cells and macrophages, was completed to gain a deeper understanding of the immuno-pathogenesis of VUE. GeoMx Digital Spatial Profiling was performed on placental tissue from 4 high grade VUE cases and 4 controls with no underlying pathology. Placental tissues were fluorescently labeled with CD3 and CD68 antibodies and oligo-conjugated antibodies against 48 protein targets. Overall, T cells in VUE exhibited upregulated markers of activation, memory, and antigen experience compared to controls and were altered based on placental location (villi vs. decidua). Additionally, villous macrophages in VUE upregulated costimulatory and major histocompatibility complex class I and II molecules compared to controls and macrophage subtypes in the decidua. Data herein provides new mechanistic insights into T cell and macrophage biology in VUE which contribute to this abnormal immune response to pregnancy.

## Introduction

Villitis of unknown etiology (VUE) is an inflammatory pathology of the placenta resulting in necrosis, sclerosis, and fibrosis of the fetal villi^1^. The inflammatory infiltrate that defines VUE is composed predominately of maternal T cells, with an accompanying accumulation of fetal macrophages in the villous stroma^2–4^. VUE is diagnosed in 5–15% of all third trimester placentae with a risk of recurrence in subsequent pregnancies ranging from 30 to 55%^5–7^. This pathology can be classified as low- or high-grade depending on the number of villi involved, with low-grade VUE generally resulting in a live birth whereas high-grade VUE is more likely associated with poor obstetric and neonatal outcomes^7,8^. These include an increased prevalence of fetal growth restriction (FGR), risk of stillbirth, and preterm birth^9–13^. There is also an increased risk of neurologic impairment with VUE associated infants, which may not be recognized until years after birth^14,15^. In cases of recurrence, severity of VUE tends to increase in subsequent pregnancies, further elevating the risk of adverse outcomes^11,16^. Clinically, women with autoimmune diseases and those that have undergone in vitro fertilization (IVF) with a donor oocyte have a higher risk of developing VUE^17–19^. Currently, there are no methods for diagnosing VUE prior to delivery, making it challenging to clinically manage this etiology and prevent negative outcomes.

To develop strategies that can predict and prevent VUE in utero*,* and especially its recurrence, it is necessary to uncover the underlying mechanisms of this inflammatory disease. There are two main hypotheses behind the origin of VUE. The first is that VUE is actually a form of infectious villitis, resulting from an undiagnosed infection, while the second posits that VUE represents an immunological rejection response to the semi-allogenic fetus^7^. While there have been reports of viral sequences detected in cases of VUE, definitive data identifying cryptic viral infection as a cause of all, or a subset of VUE cases, has been forthcoming^20^. Indeed, utilizing T cell receptor spectratyping, we have previously shown that T cells present in VUE do not recognize common viral epitopes unlike infectious villitis^2^. Further, while chemokines like CXCL10 are normally expressed at low physiologic levels in the placenta^21^, during VUE there is an upregulation of T cell attracting chemokines, CXCL10, CXCL9 and CXCL11^22,23^. There is also upregulation of intercellular adhesion molecule-1 (ICAM-1) on trophoblasts cells in placentae diagnosed with VUE at sites with high leukocyte infiltration^24–26^. ICAM-1 is normally expressed on vascular endothelial cells and is important for leukocyte adhesion, as well as for promoting transmigration of cells across the endothelium^27,28^. In addition, there is increased C4d deposition in VUE placenta as compared to controls and infectious villitis cases^29,30^, which has been shown to be indicative of antibody mediated rejection following tissue transplant^31^. Lastly, we have previously demonstrated that there is decreased expression of inhibitory receptor PD-L1 on the syncytiotrophoblast cells, which may promote maternal T cell targeting^32^. Overall, there is strong evidence to support the hypothesis that VUE reflects an aberrant immune response likely directed against the haploidentical fetus.

While VUE was first described in the 1970s, and many studies associating clinical outcomes with this diagnosis have been reported, numerous questions remain regarding the true immuno-pathogenesis of this inflammatory etiology. Our objective was to phenotypically and functionally define the major immune cell types involved in VUE, specifically maternal T cells and fetal macrophages. We employed spatial proteomics to elucidate phenotypic and functional differences in CD3 T cells and CD68 macrophages in VUE compared to controls. In addition, we addressed how the phenotypes of these immune cells were altered based on placental location. These data can help to elucidate the mechanisms behind inflammatory responses driving VUE.

## Results

### Patient demographics

**Figure 1:**
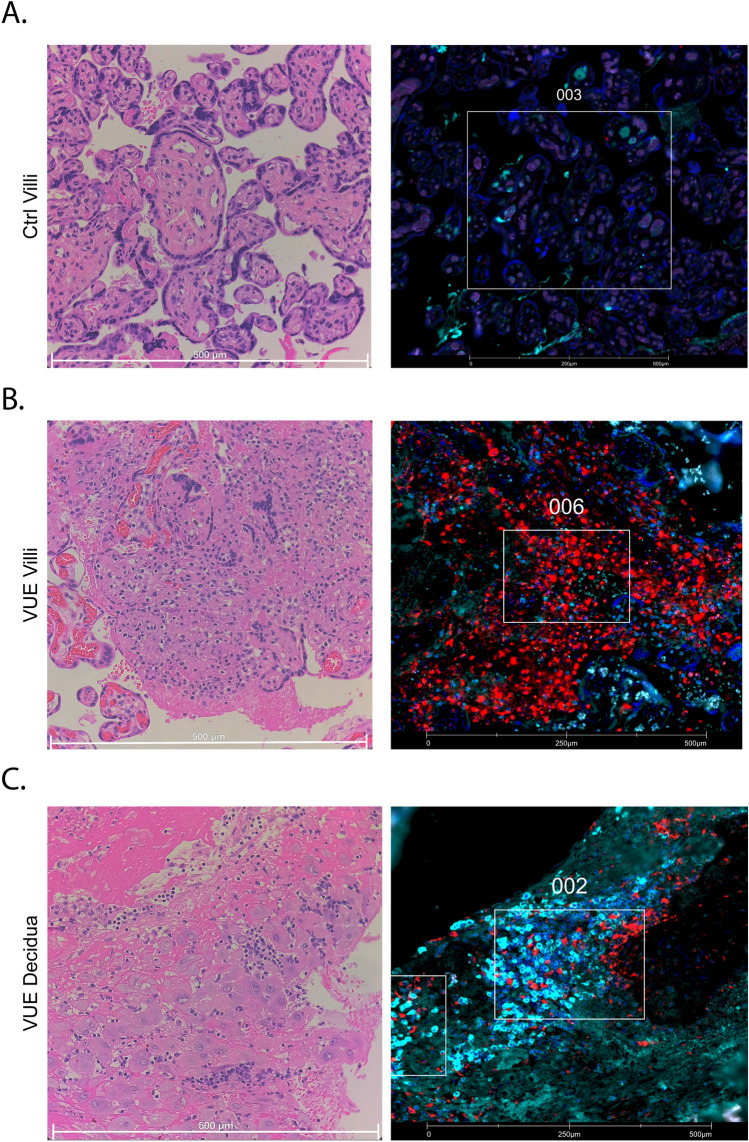
Representative histological and immunofluorescent (IF) images of control and VUE placentae. For IF images, CD3+ cells are shown in red, CD68+ cells are shown in cyan, and nuclei (SYTO13) shown in blue. (**A**) Hematoxylin and eosin (H&E) (left) and IF (right) of control (ctrl) villi. (**B**) H&E (left) and IF (right) of VUE villi. (**C**) H&E (left) and IF (right) of VUE decidua.

Eight subjects were included in this study, 4 diagnosed with high-grade VUE and 4 controls with no identifiable placental pathology of significance. Patient demographics are listed in Table 1. There were no significant differences based on maternal age, gestational age at delivery, body mass index, gravidity, or fetal sex between patients with VUE and controls. The birthweight of neonates in the VUE group were significantly smaller than that of controls (p = 0.03). Regions of interest and areas of illumination (ROIs and AOIs, respectively) were selected based on the presence of immune cell infiltration and placental location (i.e., villi vs. decidua). For each tissue, 6 ROIs were selected which corresponded to 12 AOIs. To assess placental location, 4 of 6 ROIs were selected from villous tissue and 2 were selected from decidua for each slide. For controls, there was very little decidual tissue present (n=2) so further analysis of this region could not be pursued (Supplemental Table 1). Representative histological and immunofluorescent images for control villi, VUE villi, and VUE decidua are shown in Fig. 1A–C. To understand global changes in VUE and control placentae, we performed a linear mixed modeling analysis of T cells and macrophages, including all ROIs across placental compartments, which revealed that T cells in VUE upregulated 8 proteins and downregulated 2 proteins compared to controls (Supplementary Fig. 1A). Macrophages across the placenta in VUE upregulated 7 proteins and downregulated 1 target compared to controls (Supplementary Fig. 1B). These global differences lead us to further analyze changes in VUE and control placentae based on placental location.

**Table 1:** Patient demographics.

	Control	VUE	P-value
Age (years)	38 (36–45)	37.5 (22–42)	0.6857
Gestational age (weeks)	40 (39.2–40.2)	39.6 (34.3–40.2)	0.5715
Gravidity	3 (2–5)	1.5 (1–3)	0.6734
BMI	21 (21–26)	26 (22–40)	0.5143
Birthweight (g)	3655 (3360–4110)	3175 (2680–3350)	0.0286*
Fetal sex (%female)	25% (1/4)	50% (2/4)	0.9167

Data presented as median with ranges or as percentages with proportion (fetal sex). P-values were identified with either a Mann–Whitney test or a Chi-square analysis, significance indicated with *. *BMI* body mass index.

### Unique T cell phenotypes in VUE villi

**Figure 2:**
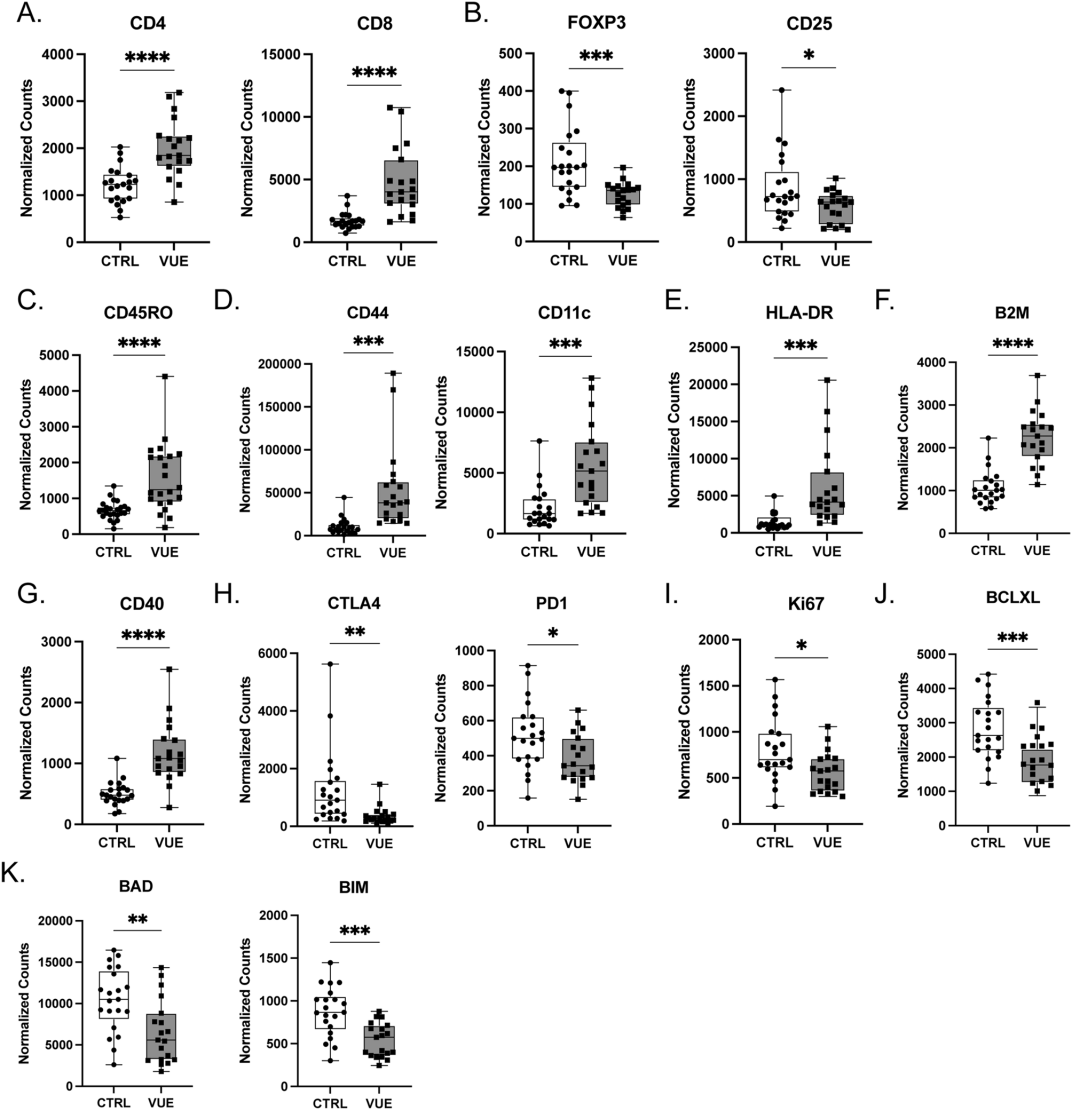
Unique T cell phenotypes in the villi of VUE placentae. Normalized counts of CD3+ T cell subsets in the villi of VUE and control (ctrl) placentae: (**A**) CD4 and CD8 T cells; (**B**) T regulatory cell markers FoxP3 and CD25; and (**C**) CD45RO Memory T cells. (**D**) Antigen recognition receptors CD44 and CD11c; (**E**) Activation marker HLA-DR; (**F**) B2M; and (**G**) CD40. (**H**) Inhibitory receptors CTLA4 and PD-1; (**I**) proliferation via Ki67 positivity; (**J**) Anti-apoptotic factor BCLXL; and (**K**) Apoptosis proteins BAD and BIM. Data is represented as median with ranges (n = 19 VUE, n = 21 ctrl). Significance determined by unpaired t test and denoted as *p ≤ 0.05, **p ≤ 0.01, ***p ≤ 0.001, ****p ≤ 0.0001.

To characterize the phenotype and function of T cells in VUE villi, we compared the proteomic profiles of CD3+ T cells across VUE villous tissue compared to villous tissue of controls. All data are presented as mean normalized counts. As per the definition, maternal T cells must be seen infiltrating into fetal villi during VUE pathology^1–4,33^. Therefore, in VUE cases only ROIs where this infiltration was present were chosen for GeoMx analysis. For control villous tissue, we would not expect T cells to be present within the chorionic villi; thus, ROIs in which T cells were mainly present in the intervillous space were chosen. As expected, there were significantly more CD4+ (2007 vs. 1221; p ≤ 0.0001) and CD8+ (4766 vs. 1731; p ≤ 0.0001) T cells in placentae with VUE compared to controls, respectively (Fig. 2A). Conversely, there were fewer T regulatory cells (Tregs) in VUE defined by an overall decrease in FoxP3+ (125.2 vs. 212.2; p = 0.0003) and CD25+ cells (565.2 vs. 866.5; p = 0.0286; Fig. 2B). To further characterize the function of the T cells identified in VUE, we evaluated different activation markers indicative of whether cells had encountered and responded to antigen. Specifically, we observed a significant increase in CD45RO+ T memory cells during VUE compared to controls (1565 vs. 667.4; p ≤ 0.0001; Fig. 2C). Also, we noted that antigen recognition receptors CD11c and CD44 were significantly increased on T cells in VUE compared to controls (5629 vs 2142; p = 0.0002 and 53,968 vs 10,624; p = 0.0003 respectively; Fig. 2D). Activation marker HLA-DR was also highly increased on T cells in VUE compared to controls (6200 vs 1516; p = 0.0004; Fig. 2E), as well as Beta-2-microglobulin (B2M) on VUE T cells compared to controls (2242 vs 1073; p < 0.0001; Fig. 2F). Additionally, we noted that CD40 was significantly upregulated on T cells in VUE compared to controls (1172 vs 505.0; p ≤ 0.0001; Fig. 2G), which promotes T cell differentiation into a memory phenotype. Conversely, inhibitory receptors CTLA4 and PD-1 were significantly decreased on T cells during VUE compared to controls (366.2 vs 1271; p = 0.0060 and 380.4 vs 515.4; p = 0.0154 respectively; Fig. 2H). T cells in VUE exhibited reduced proliferative capacity (Ki67) compared to controls (564.9 vs. 801.4; p = 0.0133; Fig. 2I). Additionally, T cells in VUE had decreased abundance of anti-apoptotic factor BCLXL (1859 vs 2838; p = 0.0004; Fig. 2J) suggesting decreased survival. Lastly, apoptotic markers BAD and BIM were significantly reduced on T cells in VUE compared to controls (6553 vs 10,453; p = 0.0028 and 541.8 vs 875.0; p = 0.0001, respectively; Fig. 2K). Altogether, these data demonstrate that CD4 and CD8 T cells are increased in the villi of VUE placentae while Tregs are decreased. Further, it reveals that T cells present in VUE villi exhibit an activated and antigen experienced phenotype with decreased abundance of inhibitory receptors.

### Fetal macrophage phenotypes in VUE villous tissue

**Figure 3:**
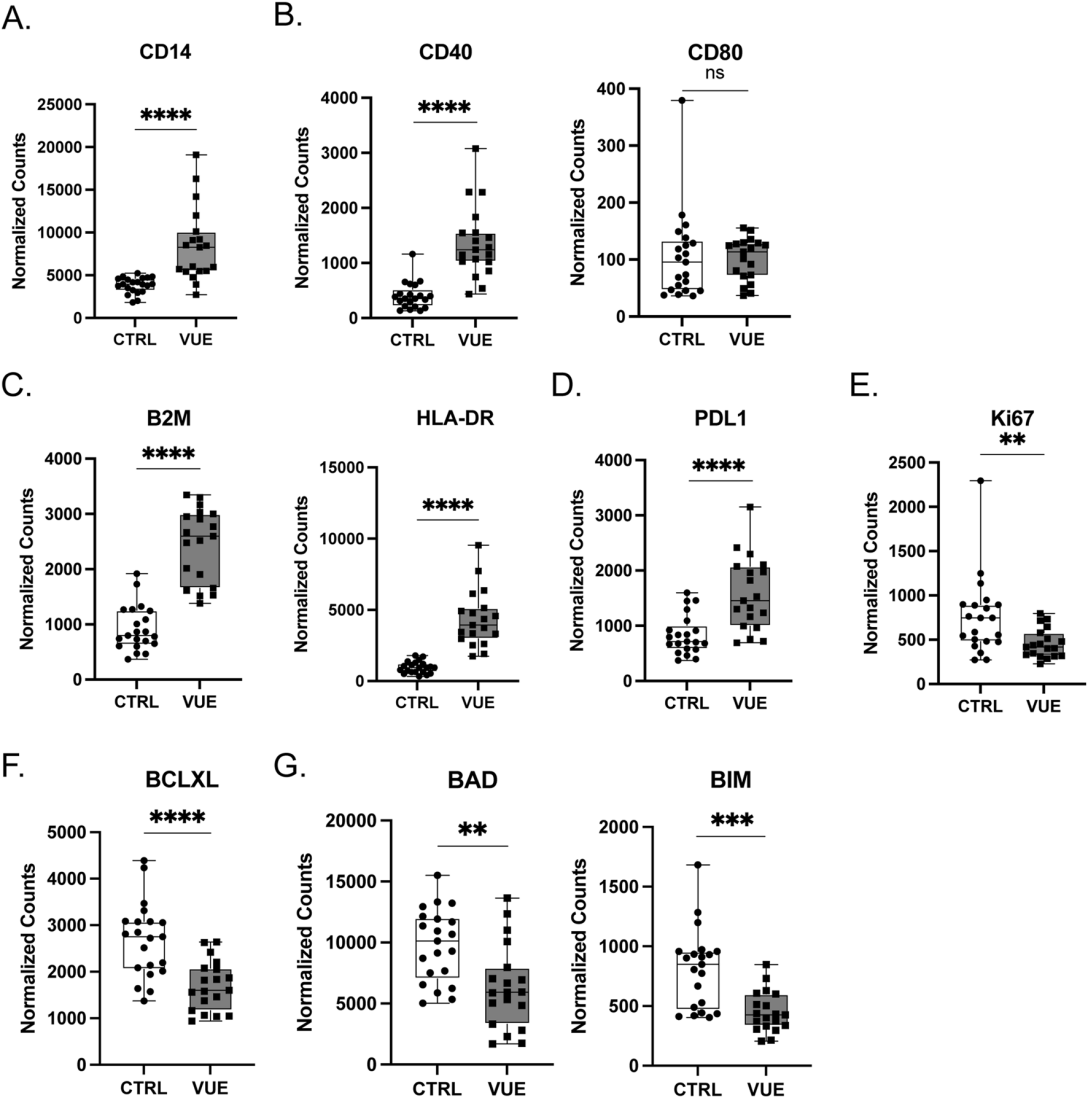
Fetal macrophage phenotypes in VUE villous tissue. Normalized counts of CD68+ macrophages in the villous tissue of VUE placenta compared to control (ctrl) villous tissue: (**A**) CD14 on macrophages; (**B**) costimulatory molecules CD40 and CD80; (**C**) B2M and HLA-DR; (**D**) PDL1; (**E**) Ki67. (**F**) Anti-apoptotic factor BCLXL; and (**G**) Apoptotic markers BAD and BIM. Data is represented as median with ranges (n = 19 VUE, n = 21 ctrl). Significance determined by unpaired t test and denoted as *p ≤ 0.05, **p ≤ 0.01, ***p ≤ 0.001, ****p ≤ 0.0001.

Macrophages present in the chorionic villi of placentae are fetal in origin and termed Hofbauer cells^34^. Fetal macrophages are more abundant in the villi of VUE placentae^3,34^, therefore we assessed villous macrophage phenotypes, defined by CD68 staining, in VUE compared to control placentae. In VUE, there was significant upregulation of CD14 in villous macrophages compared to controls (Fig. 3A). Fetal macrophages in VUE significantly upregulated costimulatory molecule CD40 (1367 vs. 400.9; p ≤ 0.0001), but not CD80 (p = 0.8422; Fig. 3B). Also, there was increased abundance of both MHC class I (B2M) as well as MHC class II (HLA-DR) molecules in VUE macrophages compared to controls (2439 vs 928.7; p ≤ 0.0001 and 4371 vs. 958.7; p ≤ 0.0001 respectively; Fig. 3C). Lastly, in VUE there was upregulation of PD-L1 (1576 vs 835.0; p ≤ 0.0001; Fig. 3D), a downregulation of Ki67 (458.4 vs. 757.1; p = 0.0087; Fig. 3E), a significant decrease in anti-apoptotic marker BCLXL (1704 vs 2681; p < 0.0001; Fig. 3F), and apoptotic markers BAD and BIM in fetal macrophages (6341 vs 9770; p = 0.0017 and 450.9 vs 807.7; p = 0.0002, and respectively; Fig. 3G). Overall, this supports previous data indicating that fetal macrophages are increased in VUE. Additionally, our data suggests fetal macrophages in villous tissue could be involved in antigen presentation during VUE pathogenesis as demonstrated by increased abundance of CD40 and MHC class I and II.

### T cell phenotypes based on tissue location during VUE

**Figure 4:**
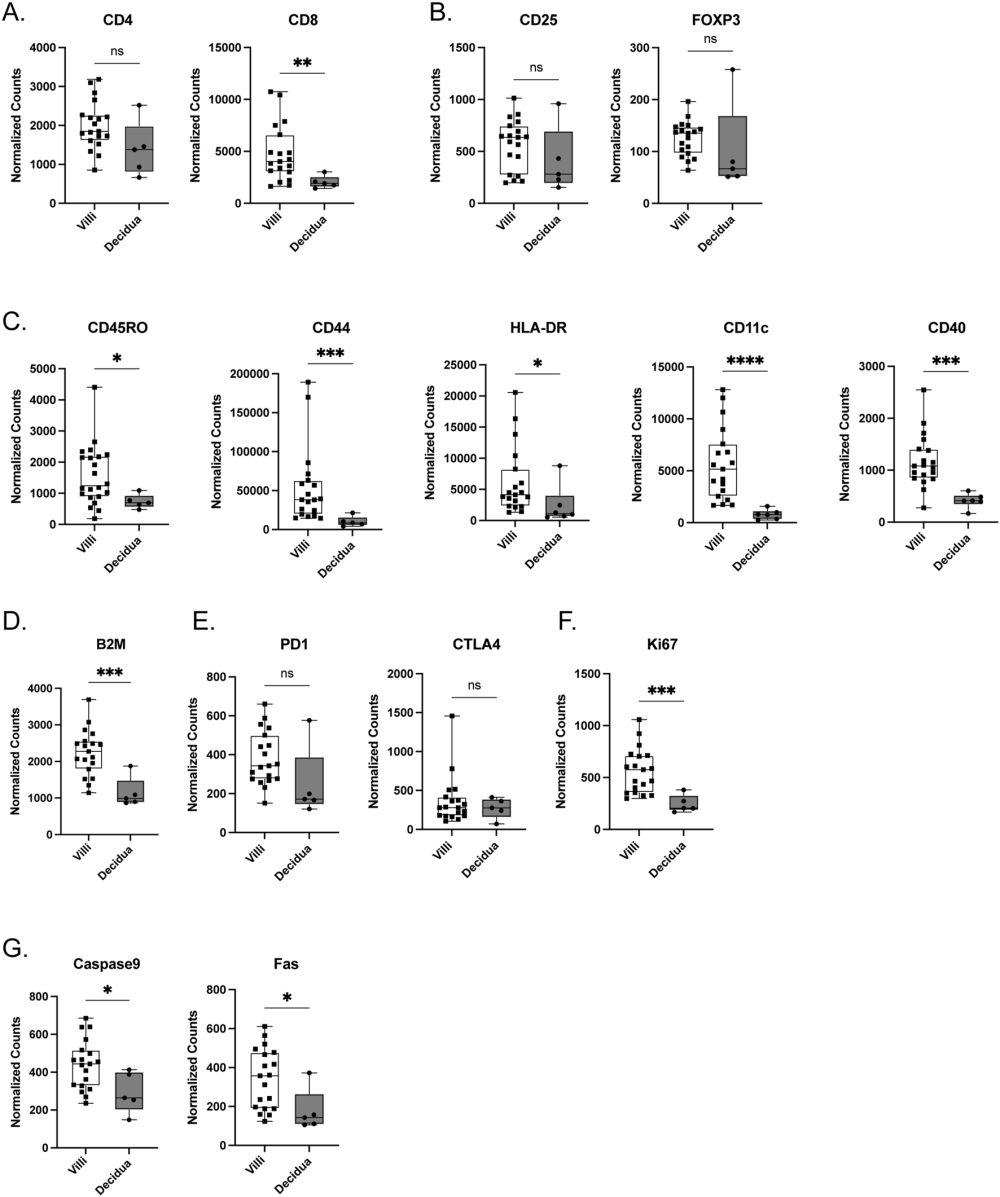
Changes in CD3+ T cell phenotypes by placental location during VUE. Normalized counts of proteins in the villi compared to decidua of VUE placentae: (**A**) CD4 and CD8; (**B**) CD25 and FoxP3; (**C**) activation and antigen recognition targets CD45RO, CD44, HLA-DR, CD11c, and CD40. (**D**) B2M; and. (**E**) inhibitory receptors CTLA4 and PD-1. (**F**) Ki67 proliferation; and (**G**) cell death markers Caspase9 and Fas. Data is represented as median with ranges (n = 19 villi, n = 5 decidua). Significance determined by Mann–Whitney test and denoted as *p ≤ 0.05, **p ≤ 0.01, ***p ≤ 0.001, ****p ≤ 0.0001.

Next, we aimed to address whether T cells and macrophages during VUE exhibited altered phenotypes based on location, specifically in the villous stroma versus decidua. All data is represented as median normalized counts. When evaluating T cell subtypes, no significant differences in the abundance of CD4 T cells (p = 0.0750) based on location were noted, however CD8 T cells were significantly increased in villous tissue of VUE placentae compared to decidua (4014 vs 1903; p = 0.0053; Fig. 4A). There was also no difference in abundance of Tregs in villous tissue compared to decidua in VUE, as determined by comparable expression of CD25 and Foxp3 on CD3 T cells at each location (p = 0.2647 and p = 0.0632 respectively; Fig. 4B). We again compared activation markers in the villous tissue of VUE placenta compared to decidua to understand the function of these cells. Within villous tissue, VUE T cells have a higher abundance of CD45RO (1244 vs 695.4; p = 0.0333), CD44 (38,279 vs 8601; p = 0.0009), HLA-DR (4125 vs. 1117; p = 0.00171), CD11c (5151 vs 770.5; p ≤ 0.0001), and CD40 (1080 vs 411.8; p = 0.0002) compared to T cells in the decidua (Fig. 4C). Additionally, villous infiltrated T cells showed a significant increase in B2M compared to T cells in decidua (2276 vs 987; p = 0.0009; Fig. 4D). When looking at inhibitory receptors PD-1 and CTLA-4, we noted no significant difference in abundance based on placental location (p = 0.0529 and p = 0.7831, respectively; Fig. 4E); however, PD-1 showed a positive trend towards significance in placental villi. Lastly, we observed that T cells in villous tissue during VUE exhibit higher proliferative capacity compared to decidua as defined by Ki67 (575.8 vs. 204.8; p = 0.0009; Fig. 4F), as well as upregulation of cell death markers Caspase 9 and Fas (443.7 vs 264.7; p = 0.0296 and 357.8 vs 143.0; p = 0.0152 respectively; Fig. 4G). Taken together, these data indicate that T cells in VUE are altered based on placental location and are more activated in villi compared to decidual tissue.

### Macrophage comparisons based on location within placental compartments

**Figure 5:**
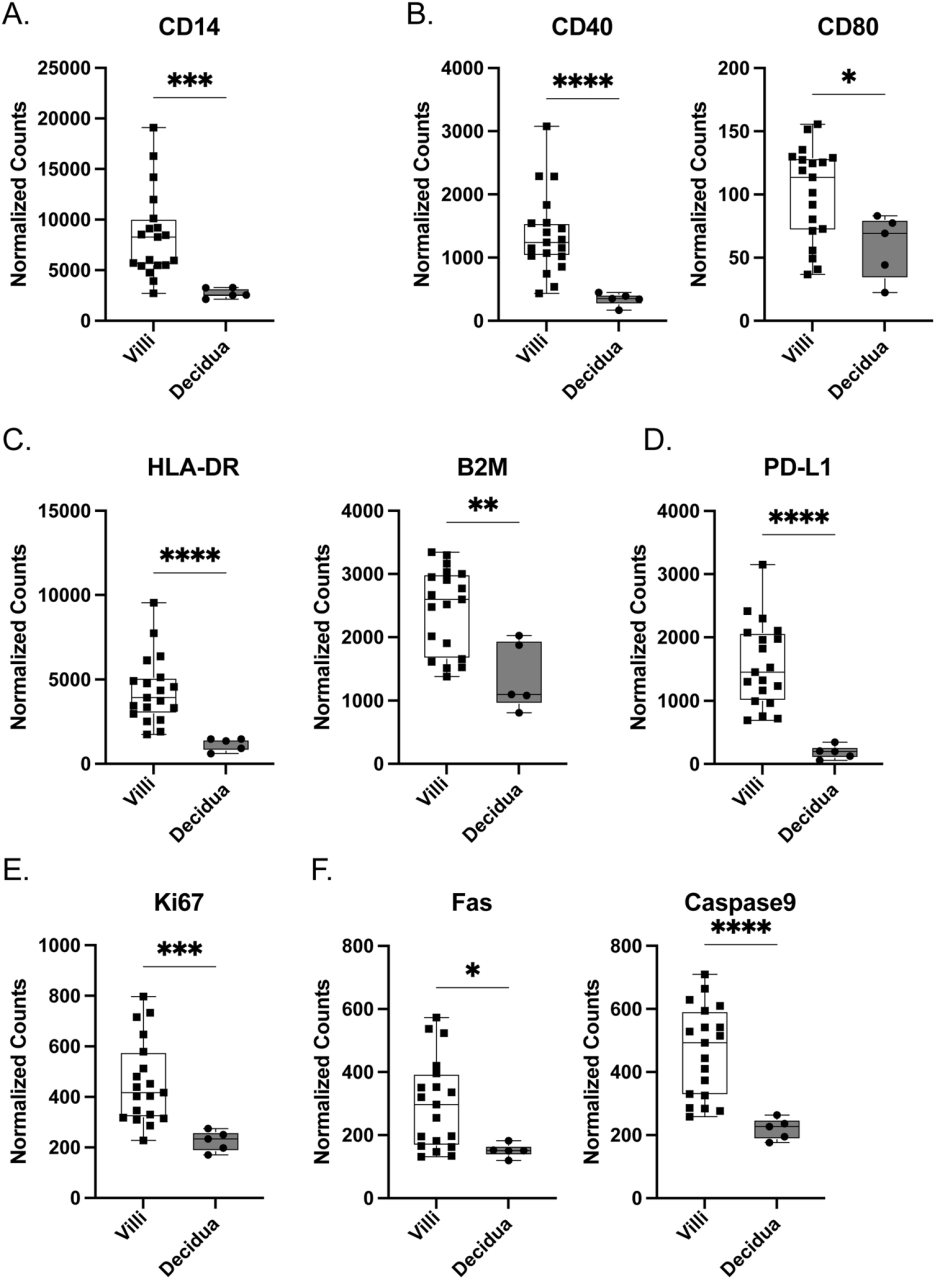
Macrophage phenotypes differ within villi and decidual compartments in VUE placenta. Normalized counts of CD68+ macrophage proteins in the villi compared to decidua of VUE placentae: (**A**) CD14; (**B**) costimulatory molecules CD40 and CD80; (**C**) HLA-DR and B2M; (**D**) PD-L1; (**E**) Ki67 proliferation; and (**F**) cell death markers Fas and Caspase 9. Data is represented as median with ranges (n = 19 villi, n = 5 decidua). Significance determined by Mann–Whitney test and denoted as *p ≤ 0.05, **p ≤ 0.01, ***p ≤ 0.001, ****p ≤ 0.0001.

We examined how macrophage phenotypes are altered in VUE based on placental location, in the villi or decidua. In general, macrophages in villous tissue are mainly fetal in origin, whereas macrophages in decidua are maternally derived^3^. Focusing on expression of CD14, we observed increased villous macrophages compared to decidual macrophages during VUE (8278 vs. 2562; p = 0.0002; Fig. 5A). Regarding costimulatory molecules, fetal macrophages in villous tissue significantly upregulated CD40 and CD80 compared to maternal macrophages in decidua (1243 vs. 350.2; p ≤ 0.0001 and 113.5 vs. 69.30 p = 0.0439 respectively; Fig. 5B). In fetal villous macrophages, we noted significant upregulation of MHC class I (2597 vs. 1098; p = 0.0093) and MHC class II (3930 vs 1354; p ≤ 0.0001) molecules, as inferred by B2M and HLA-DR respectively (Fig. 5C). Fetal macrophages in the VUE setting also showed an increased abundance of PD-L1 (1454 vs. 198.4; p ≤ 0.0001; Fig. 5D) and Ki67 (416.7 vs. 233.5; p = 0.0003; Fig. 5E) compared to maternal decidual macrophages. Similar differences were observed in cell death markers Caspase 9 and Fas between fetal and decidual macrophages (493.3 vs 227.2; p < 0.0001; Fig. 5F). Together, these data demonstrates that macrophages in the villi are more abundant and upregulate costimulatory molecules and proliferation markers compared to macrophages in the decidua in VUE.

## Discussion

VUE is defined by the infiltration of maternal T cells and accumulation of fetal macrophages in the villous stroma. In this study, we identified phenotypic and functional differences between these two cell types in VUE compared to control placentae. Also, we demonstrated phenotypic differences in T cells and macrophages based on spatial location within the placenta. We noted that VUE T cells are more antigen experienced, activated, and have downregulated inhibitory receptors, proliferative signals, as well as apoptotic markers compared to T cells in control placentae. Macrophages in VUE significantly upregulated costimulatory receptors, PD-L1 checkpoint receptors, and MHC molecules. When analyzing spatial differences within VUE placentae, we observed that maternal T cells in villous tissue were also more activated, antigen experienced, and exhibited upregulated proliferative and apoptotic markers compared to maternal T cells in the decidua. Lastly, we found that fetal macrophages were more abundant and upregulated costimulatory and MHC class I and II molecules in the villi as compared to decidual macrophages during VUE.

It has been hypothesized that VUE represents a maternal rejection response to the haploidentical fetus^7^. Memory CD4 and CD8 T cells play a major role in graft rejection, and when looking at organs following immune rejection, there is a significant increase in memory CD8 T cells expressing CD45RO^35,36^. While CD8 T cells are playing a major role in the pathophysiology of organ rejection, it is not without CD4 T cell participation, as memory CD4 T cells have been shown to mediate rejection by activating CD8 T cells^37,38^. Our data demonstrates that T cells in VUE placentae exhibit phenotypic similarities to those participating in organ rejection responses. Specifically, there is a significant infiltration of CD45RO memory T cell populations in VUE, and an increase in CD44, a receptor upregulated on tissue resident memory T cells^39^. Further, we observed an increase in CD40 on T cells in VUE which may suggest that CD4 T cells can provide costimulatory signals to CD8 T cells, as has been previously shown^40^. There is also an increase in CD11c+ T cells in VUE. CD8+CD11c+ T cells proliferate after exposure to antigen, produce high levels of interferon gamma (IFNγ), and increase cytotoxicity^41,42^. During normal pregnancy, cytotoxic T cells recognizing fetal antigen undergo clonal deletion to protect the fetal allograft^43^; however, during VUE our data suggests these T cell subsets have been exposed to antigen, are activated, and have generated a memory response. We hypothesize that this could lead to increased IFNγ production to further promote cytotoxicity and trophoblast necrosis, although this would require additional work to be confirmed.

In the pathophysiology of graft versus host disease (GVHD), increased inflammation after organ transplantation is associated with presentation of alloantigen to donor T cells which proliferate in response to non-self antigen^44–46^. T cells can upregulate survival factors to prolong their cytotoxic ability resulting in more severe GVHD^47^. While these immune cells undergo a period of rapid proliferation and cytotoxicity, afterwards a period of increased activation induced cell death ensues^48^. We have previously shown that in high-grade VUE placentae there is significant upregulation of genes involved in T cell trafficking and the pathways most significantly impacted by VUE were those involved in GVHD and allo-rejection^22^. When evaluating location specific differences, our results demonstrate that during VUE both T cells and macrophages in villous tissue not only increase proliferative and survival factors, but also exhibit increased markers of cell death. This, in combination with the antigen experienced phenotype of T cells present in VUE, provides additional evidence that VUE and GVHD have similar underlying mechanisms.

During organ rejection responses, T cells recognize alloantigen through the direct, indirect, or semi-direct pathways. Briefly, in the direct pathway of allorecognition donor antigen presenting cells (APC) present alloantigen to recipient T cells whereas in the indirect pathway, alloantigen is processed by recipient APCs and presented to recipient T cells^49^. In the semi-direct pathway, donor MHC is acquired by recipient APCs and presented to recipient T cells^49^. In our study, we have found that in placentae diagnosed with VUE, fetal macrophages are significantly increased in the villi compared to controls. These fetal macrophages have upregulated MHC class I and II, and costimulatory molecules CD40 and CD80. This data suggests that through upregulation of MHC, fetal macrophages could directly present alloantigen to T cells in the villous tissue of VUE placentae, similar to what is seen in organ rejection via direct allorecognition. Additionally, with the significant increase in CD40 and CD80, these fetal macrophages may further propagate T cell activation through binding to costimulatory ligands on the surface of T cells.

Characterizing the immune landscape at the maternal fetal interface and within the placenta has been challenging until more recently due to the development of multi-parameter technologies. For example, high dimensional flow cytometry coupled with dimensionality reduction and automated clustering, when used in conjunction with functional studies, has been utilized as a method to identify unique immune cell phenotypes present in the decidua^50,51^. Single cell sequencing technology has provided in depth characterization of the cells and pathways utilized by reproductive tissues during normal pregnancy, preterm birth, and throughout the first trimester^52–55^. These data began to highlight the possibility that there are unique immune cell phenotypes based on placental location, which are lost during tissue digestion. Thus, spatial multi-omics approaches, which allow for proteomic or transcriptomic profiling by ROI, are now being used to create a comprehensive map of the human reproductive tissues and highlight changes in cell signaling networks by location^55^. A recent study of the early maternal fetal interface, which was built on the knowledge gained by the technologies highlighted above, now provides a spatially resolved multi-omics single cell atlas defining trophoblast differentiation during the first trimester of pregnancy^54^. Our work additionally highlights the importance of considering location specific differences in cell phenotypes within complex tissues, such as the placenta.

While this study provides new insights into T cell and macrophage phenotypes critical to VUE pathogenesis, there were some limitations to the current study. Overall, we compared a small number of cases and controls thus future studies should include a larger cohort to confirm our findings. Although it has been reported that most T cells present in VUE placentae are maternal in origin, and that the macrophages present in the villous tissue are fetal derived^2–4^, we did not specifically confirm this. Additionally, we were unable to compare proteomic changes in the decidua of VUE compared to that of control placenta as there were not enough decidual regions present in the latter to make appropriate statistical comparisons. Lastly, our proteomic analysis was limited by the number of targets we were able to evaluate in each tissue. A deeper characterization of the immune cells present is needed to better understand the pathobiology of VUE.

In conclusion, this study provides new insights into the two major cell populations involved in VUE. Overall, T cells present in the villous tissue of VUE placentae demonstrate similar activation and antigen experienced phenotypes to T cells participating in organ rejection responses. Macrophages in VUE have upregulated MHC and costimulatory molecules, which may indicate that they can directly present alloantigens to T cells during VUE. Further functional studies into these mechanisms will be crucial to understand the pathophysiology of this disease in order to develop future strategies to diagnose and treat VUE during pregnancy.

## Methods and materials

### Patient characteristics

The study was approved by the Mayo Clinic Institutional Review Board (IRB# 20-012379). Research performed during this study followed strict regulations as per our institutional guidelines. Only participants who provided informed consent for use of their clinical residual tissues were included in this study and confirmed by Minnesota Research Authorization status. Clinical residual formalin fixed paraffin embedded (FFPE) tissues were obtained from four subjects diagnosed with high-grade VUE and four subjects having no placental pathology. Samples were excluded if they showed co-occurring inflammatory pathologies such as chronic histiocytic intervillositis and massive perivillous fibrin deposition to ensure we do not have infiltrating maternal histiocytes confounded the results of the cases we chose. The objective of this study was to compare differences in immune cell phenotypes, specifically CD3 and CD68, in placentae with VUE versus controls, as well as identify differences in cell profiles based on location within the placenta using spatial proteomic analysis.

### GeoMx digital spatial profiling

The following methods for GeoMx Digital Spatial Profiling have been previously described^56–58^ (NanoString, Seattle, WA). Briefly, 5-μm sections of FFPE placental tissues from either VUE or controls were baked onto glass slides, followed by tissue deparaffinization and rehydration before antigen retrieval was performed. Tissue samples were blocked and slides were incubated with primary conjugated antibodies overnight. Primary antibodies used for defining cell morphology included anti-CD3-AF594 (5 μg/mL, UMAB54, OriGene, Rockville, MD), anti-CD68-AF647 (0.50 μg/mL, KP1, Santa Cruz Biotechnology, Dallas, TX), and anti-SYTO13-FITC (NanoString). In addition, the slides were stained with oligo-conjugated antibodies that assessed 48 unique protein targets specific to immune cell profiling, cell typing, activation, cell death and housekeeping proteins. The slides were scanned with the GeoMx Digital Spatial Profiler (NanoString) and regions of interest (ROI) were selected based on the 3 fluorescent markers as well as by location. Within each ROI, areas of illumination (AOIs) were captured based on CD3 SYTO13 positivity and CD68 SYTO13 positivity. 6 ROIs and 12 AOIs were selected per tissue from regions of the villi and decidua. The oligos from the oligo-conjugated antibodies were then cleaved by UV light exposure from each AOI and collected in separate wells of a 96 well plate. Oligos were then hybridized and counted using the nCounter (NanoString).

### Statistics

Patient demographics were presented as medians with ranges and analyzed with a Mann–Whitney test for continuous variables and a chi square analysis for categorical data. For the DSP proteomics data, quality control and normalization strategies were followed as recommended by the manufacturer. Specifically, data normalization was completed using housekeeping genes (GAPDH, S6, and Histone H3) and a background correction was applied using IgG isotype controls (Rabbit IgG, Mouse IgG1, Mouse IgG2a). Total counts for each specific protein were obtained and compared using a linear mixed model analysis with a Bonferroni correction for multiple comparisons. Comparisons between AOIs based on diagnosis and location were calculated using either an unpaired *t* test or Mann–Whitney test with GraphPad Prism software version 10.0.2 (GraphPad Software, San Diego, CA).

### Supplementary Information

Supplementary Figure 1.

Supplementary Table 1.

## Data Availability

All raw data will be shared as either a .RCC or .xlsx file upon reasonable request to the corresponding author.
