# Supplementary material for: Spatial proteomics reveals phenotypic and functional differences in T cell and macrophage subsets during villitis of unknown etiology
Source: Sci Rep. 2024 Jan 9;14:914. doi: 10.1038/s41598-024-51545-2 (PMC10776790; doi:10.1038/s41598-024-51545-2)
Supplement: Supplementary file 1 — Supplementary Figure 1. [file 41598_2024_51545_MOESM1_ESM.docx]

**
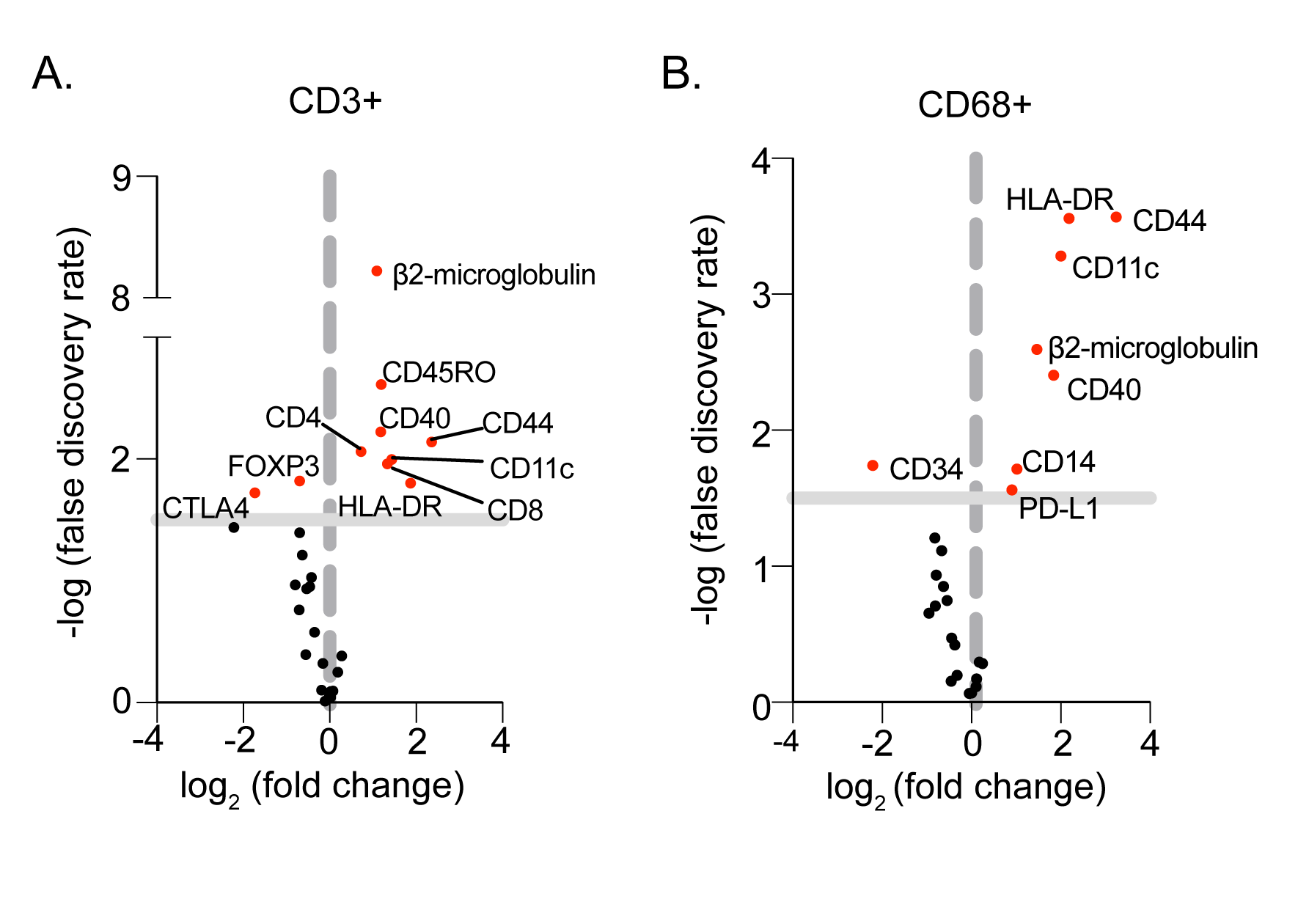
Supplementary Figure 1. Total proteomic differences in VUE placentae.** (A) Volcano plot of proteins significant upregulated and downregulated in CD3+ cells in VUE compared to controls; (B) Volcano plot of proteins significantly altered in CD68+ cells in VUE compared to controls.
