# Supplementary material for: Spatial proteomics reveals phenotypic and functional differences in T cell and macrophage subsets during villitis of unknown etiology
Source: Sci Rep. 2024 Jan 9;14:914. doi: 10.1038/s41598-024-51545-2 (PMC10776790; doi:10.1038/s41598-024-51545-2)
Supplement: Supplementary file 2 — Supplementary Table 1. [file 41598_2024_51545_MOESM2_ESM.docx]

**Supplementary Table 1.** Average normalized counts of all 48 unique proteins + standard deviation

| Target Name | CONTROL VILLI | | Control Decidua | | VUE Villi | | VUE Decidua | |
| --- | --- | --- | --- | --- | --- | --- | --- | --- |
|  | ***CD3*** | ***CD68*** | ***CD3*** | ***CD68*** | ***CD3*** | ***CD68*** | ***CD3*** | ***CD68*** |
| *CD44* | 10090.1 (4624.7) | 4382.9 (1960.3) | 1159.5 (396.4) | 6306.2 (3795.4) | 57500.1 (48161.4) | 43004.3 (13083.8) | 9269.4 (1380) | 14743.1 (10173.6) |
| *CD25* | 828.7 (403.6) | 809.3 (522.3) | 275.7 (16.4) | 391.7 (212.9) | 586.7 (234.4) | 512.9 (180.9) | 279.9 (140.6) | 308.9 (173.5) |
| *CD27* | 259 (39.1) | 214.8 (104.3) | 79.5 (10.6) | 145.8 (78.5) | 337.9 (144.1) | 255.6 (110.9) | 184.9 (46.3) | 326.7 (377.2) |
| *CD127* | 4687.3 (1304.6) | 4503 (995.3) | 3102.5 (687.5) | 3260.6 (1072.6) | 5063 (2117.5) | 4639.3 (2596.5) | 2203.9 (1323) | 2627.7 (1930.1) |
| *CD40* | 492.5 (108.4) | 386.5 (176.9) | 128.3 (37.9) | 239.4 (170.3) | 1226 (393.4) | 1377.1 (293.3) | 359.7 (68.8) | 358.1 (98.5) |
| *ICOS* | 415.9 (107.8) | 427.2 (283.5) | 93.3 (2.8) | 234 (160.4) | 453.8 (204.6) | 324.6 (113.6) | 180.1 (49.2) | 144.6 (56.2) |
| *PD-L2* | 169.5 (56) | 161.1 (61.4) | 113.3 (6.1) | 173.6 (49.2) | 181.2 (40.7) | 168.3 (33.4) | 106.3 (48.3) | 97.8 (60.3) |
| *CD80* | 103.6 (16.1) | 100.9 (56.3) | 27.5 (6.4) | 69.1 (70.7) | 91.8 (21.3) | 99.4 (16.9) | 57.3 (9.6) | 63.3 (26.3) |
| *CD66b* | 284.8 (229.5) | 168.8 (147.7) | 35.7 (23.8) | 57.2 (42) | 103 (59) | 120.3 (59.5) | 41.1 (33.4) | 20.2 (1.8) |
| *FAP-alpha* | 980.5 (68.1) | 969.3 (381) | 233.2 (34) | 624.3 (474.7) | 927.9 (272.5) | 816.7 (166.1) | 368.7 (200.4) | 372.1 (157) |
| *CD45RO* | 699.4 (124.1) | 582.9 (156.4) | 246 (11.6) | 371.6 (226.2) | 1760 (453.8) | 1516.1 (323) | 712.2 (21.3) | 600.3 (213.5) |
| *FOXP3* | 208.3 (39.7) | 163.4 (73.1) | 77.7 (10.5) | 82.8 (18.2) | 126.4 (22) | 108.9 (28.7) | 71.7 (27.5) | 66.3 (13) |
| *CD163* | 216.9 (74.1) | 213 (87) | 40.1 (6.9) | 266.1 (328.7) | 263.7 (136.1) | 290 (185.2) | 239.5 (159.4) | 229.9 (134.9) |
| *CD34* | 22124.3 (9484.6) | 13434.6 (2763.1) | 1162.5 (40.9) | 1137.5 (191.1) | 5873.2 (4834.6) | 4113.5 (2685.1) | 1264.5 (1341) | 718.9 (284.4) |
| *CD14* | 3398.3 (756.2) | 3871.7 (326.9) | 523.4 (66.2) | 1929 (1990.8) | 7758.8 (3042.5) | 8775.6 (4429.3) | 2408.4 (827.3) | 2713.5 (484.9) |
| *Ki-67* | 800.6 (77.4) | 741.8 (323.4) | 383.1 (29.5) | 673.9 (480) | 541.8 (192.1) | 452.7 (107.9) | 244.1 (85.7) | 216.1 (39.7) |
| *PD-L1* | 1060.1 (522.7) | 823.6 (338.1) | 194.3 (51.5) | 156.8 (18) | 1476 (527.3) | 1534.4 (386.1) | 243.8 (49) | 212.2 (115.7) |
| *Histone H3* | 12576.8 (6353.1) | 12756 (5499.5) | 6020 (955) | 3928.9 (463.9) | 9617.9 (435.3) | 10218.8 (1988.1) | 9496.5 (2795.6) | 8065.3 (3159.6) |
| *Ms IgG1* | 572.7 (57.1) | 587.6 (221) | 314.5 (45.3) | 508.2 (339.1) | 333.3 (109.3) | 304.7 (94.4) | 166.8 (63.4) | 148.6 (26.6) |
| *CD45* | 8833.2 (4023.8) | 5634.2 (590.5) | 2035.3 (405.1) | 5010.7 (4749.6) | 30895.3 (4216.7) | 27757.9 (5518.5) | 10256.6 (5393.2) | 14330.2 (9409.1) |
| *HLA-DR* | 1529.8 (454.2) | 969.5 (315.9) | 580.2 (39.3) | 1310.1 (946.6) | 6608.4 (5801.6) | 4345.4 (804.2) | 1455.7 (821.8) | 1213.1 (392.2) |
| *CD56* | 884.6 (109.7) | 879.7 (297.2) | 413 (21.9) | 1057.4 (414) | 576.2 (202.3) | 545.6 (173) | 754.2 (429.8) | 580 (564.3) |
| *Rb IgG* | 591.5 (94) | 638.3 (243.2) | 278.7 (53.7) | 534.1 (381.8) | 405.8 (110.8) | 367.2 (108.6) | 203.6 (80.2) | 188.8 (70.2) |
| *S6* | 10689.6 (796.9) | 11086.9 (1032.1) | 7864.9 (261.3) | 9249 (1479.2) | 10080 (2324.1) | 9142.4 (2397.9) | 10456.4 (2954.6) | 11142.7 (3687.8) |
| *PanCk* | 21009.1 (9976.3) | 20691.4 (9501.2) | 90303.8 (14108.2) | 29727.1 (21747.7) | 8461.6 (5145.6) | 6671.1 (3712.1) | 9172.1 (7015.1) | 3298.6 (4286.8) |
| *CTLA4* | 1279.7 (825.8) | 447 (284.9) | 131.2 (24.7) | 150.7 (60.1) | 362.3 (173.3) | 384.1 (188.5) | 213.7 (126.8) | 166 (90.9) |
| *Fibronectin* | 13919.1 (8468.1) | 10212.2 (688.4) | 17945.6 (13779.4) | 76279.6 (94859.3) | 6530.3 (2150.1) | 8365.8 (1439.6) | 2913.7 (1492) | 2247.2 (755.3) |
| *CD8* | 1712.3 (309.6) | 1510.1 (635) | 714.9 (21.1) | 1099.9 (719.5) | 4738.4 (2112) | 3591.2 (2025.5) | 2140.4 (749.2) | 2259.2 (1838.5) |
| *Ms IgG2a* | 280.1 (52.1) | 262.5 (103.9) | 107.9 (8) | 195.4 (149.5) | 156.5 (67.4) | 133.4 (45.8) | 76.1 (34.9) | 69.5 (35.4) |
| *GAPDH* | 24190.6 (7922.5) | 21887.3 (5779.3) | 57692 (11039.3) | 74759.9 (3160.3) | 30195.2 (5677.7) | 32438.9 (7550.5) | 29066.6 (960.4) | 33037.1 (2348.9) |
| *CD11c* | 2123.9 (391.8) | 1121.2 (212.3) | 437.8 (49.7) | 892.5 (635) | 5793 (3098.8) | 4756.4 (1393.6) | 807 (678.1) | 883.4 (735.9) |
| *CD4* | 1193.6 (285.9) | 1059.4 (360.2) | 366.6 (92.2) | 1344.4 (1444.4) | 2026.6 (290.6) | 1864.5 (142.3) | 1168.9 (338.9) | 1114.2 (395.4) |
| *CD20* | 605.4 (165.7) | 474.4 (125.6) | 269.2 (18.6) | 445.9 (238.1) | 336.6 (107.1) | 308.4 (58.7) | 173.4 (53.8) | 189.6 (88.9) |
| *CD68* | 3296.1 (557.3) | 2617.3 (1074.6) | 1194.6 (552.1) | 8690.6 (10586) | 11550.8 (14355.3) | 7746.2 (4299.8) | 1820.8 (1406.7) | 1363.9 (104.6) |
| *GZMB* | 1656.5 (534.6) | 1631.2 (842.2) | 936.9 (175.8) | 1068.6 (386) | 1723.8 (501.6) | 1569.5 (280.4) | 1251.3 (329.6) | 1148.9 (377.6) |
| *PD-1* | 515.5 (39) | 484.3 (196.7) | 264.7 (13.8) | 444.3 (298.6) | 371.1 (101.9) | 312 (88.8) | 185.8 (50.2) | 137.6 (38.5) |
| *CD3* | 673.2 (143.1) | 544.5 (270.7) | 203 (27.3) | 400.8 (286.4) | 1199.9 (288.9) | 698.3 (206.5) | 1067.1 (520.7) | 1051.4 (777.3) |
| *SMA* | 138017.3 (53764.3) | 161577.8 (66710.6) | 5830 (5224.4) | 7536.2 (7019.1) | 70892.1 (51313.2) | 76081.5 (49595.5) | 28903.9 (29216.5) | 13426.2 (4291.3) |
| *Beta-2-microglobulin* | 1071.7 (71.2) | 933.8 (276.8) | 1170.6 (191.6) | 1312.7 (795.2) | 2240.2 (141.1) | 2420 (463.5) | 1107.7 (204.3) | 1329 (541.9) |
| *BAD* | 10741.8 (2360.2) | 10011.4 (1935.4) | 7262.3 (1502.4) | 8536.6 (2153.5) | 6245.8 (3524) | 6185.6 (2962.6) | 4032.2 (2385.6) | 3410.1 (1928) |
| *BIM* | 870.8 (114.5) | 789.4 (255) | 218.4 (18) | 456.2 (235.7) | 533.5 (180.9) | 449.7 (124) | 374.8 (102.3) | 344.5 (167.7) |
| *p53* | 2330.9 (304.9) | 2662.3 (485.7) | 999.3 (67.1) | 1208 (203) | 2508.9 (1537.7) | 2269.1 (932.8) | 778.8 (508.2) | 956.1 (801.6) |
| *BCLXL* | 2820.8 (400.5) | 2641.4 (639.1) | 1051.2 (224.6) | 2017.9 (1301.1) | 1880.5 (679.2) | 1745.9 (510.3) | 1261 (662.9) | 1360.1 (918.7) |
| *BCL6* | 1443 (839.8) | 1666.3 (1671.4) | 416.2 (114.1) | 1076.9 (990) | 801.2 (372.8) | 675.3 (244.8) | 436.7 (271) | 322.4 (99.6) |
| *GZMA* | 480.9 (78) | 430.2 (251.1) | 117.1 (4.8) | 254.4 (139.1) | 408 (106) | 334.9 (106.4) | 216.9 (55.4) | 214.8 (119.8) |
| *PARP* | 976.2 (290.8) | 1164.8 (858.1) | 297 (19.3) | 532.8 (378.5) | 667.3 (191.4) | 599.2 (185.7) | 451.8 (209.2) | 372.7 (137.5) |
| *Neurofibromin* | 3466.4 (1110.1) | 3090.8 (1014.2) | 2085.4 (354) | 1684.7 (242.1) | 3455 (1427.6) | 3364.2 (1292.5) | 1781.7 (499.3) | 1791.2 (766.4) |
| *CD95/Fas* | 426.5 (55.4) | 410.1 (170.1) | 106.1 (6.4) | 228.2 (158.9) | 332.5 (125.4) | 293.4 (115.7) | 151.1 (24.2) | 151.5 (15.4) |
| *Cleaved Caspase 9* | 433.6 (46.4) | 402.3 (40.1) | 317.6 (101.6) | 436.7 (259.3) | 439.1 (104.1) | 455.6 (121.4) | 241 (85.1) | 212.4 (37) |
